# Soluble Epoxide Hydrolase Inhibitor t-AUCB Ameliorates Vascular Endothelial Dysfunction by Influencing the NF-κB/miR-155-5p/eNOS/NO/IκB Cycle in Hypertensive Rats

**DOI:** 10.3390/antiox11071372

**Published:** 2022-07-15

**Authors:** Xiaorui Wang, Wenwen Han, Yi Zhang, Yi Zong, Na Tan, Yan Zhang, Li Li, Chang Liu, Limei Liu

**Affiliations:** 1 Department of Physiology and Pathophysiology, School of Basic Medical Sciences, Peking University, Beijing 100191, China; 2011210004@bjmu.edu.cn (X.W.); 1911210009@pku.edu.cn (W.H.); 2111110083@bjmu.edu.cn (Y.Z.); 1911210056@bjmu.edu.cn (Y.Z.); tanna963@bjmu.edu.cn (N.T.); zhangy18@bjmu.edu.cn (Y.Z.); lilyby@bjmu.edu.cn (L.L.); 2111210001@stu.pku.edu.cn (C.L.); 2 Department of Integration of Chinese and Western Medicine, School of Basic Medical Sciences, Peking University, Beijing 102206, China; 3 Key Laboratory of Molecular Cardiovascular Science, Ministry of Education, Beijing 100191, China

**Keywords:** epoxyeicosatrienoic acids, endothelial dysfunction, hypertension, microRNA, reactive oxygen species

## Abstract

Epoxyeicosatrienoic acids (EETs), angiogenic mediators degraded by soluble epoxide hydrolase (sEH), have been shown to exert beneficial effects on the cardiovascular system. The current study assessed the impact of increased EETs with an sEH inhibitor, t-AUCB, on two-kidney-one-clip (2K1C)-induced renovascular endothelial dysfunction, associated with hypertension, in rats. The hypertensive rats exhibited increased systolic blood pressure, reduced renal blood flow, impaired endothelium-dependent relaxation and eNOS phosphorylation in the renal arteries, elevated ROS production in the endothelium of the renal arteries, and decreased EET levels in plasma, the renal arteries, and endothelial cells; however, t-AUCB reversed all the deleterious effects. Moreover, we found that the stimulation of AMPK/UCP2 scavenged ROS and restored endothelial function in the renal arteries of hypertensive rats undergoing therapy with t-AUCB. In addition, we were the first to reveal the potential role of miR-155-5p in the occurrence and development of vascular endothelial dysfunction in hypertension. Importantly, t-AUCB recovered NO bioavailability by regulating the NF-κB/miR-155-5p/eNOS/NO/IκB cycle after the activation of AMPK/UCP2 and the subsequent inhibition of ROS in hypertensive rat renal artery endothelial cells. This study will provide evidence for this additional new mechanism, underlying the benefits of EETs and the related agents against hypertensive vasculopathy.

## 1. Introduction

Hypertension, which is considered an important risk factor in the development of cardiovascular diseases, presents a serious threat to human life and health [1]. It appears to be associated with the occurrence and development of vascular endothelial dysfunction [2]. Endothelial dysfunction is characterized by a reduction in nitric oxide (NO) production and/or the dysfunction of NO bioavailability, due to enhanced oxidative stress caused by the excessive generation of reactive oxygen species (ROS) [3,4]. ROS can induce NF-κB activation, which is a further cause of endothelial cell injury [5]. Endothelial dysfunction, in turn, contributes to the progression of hypertension [6]. Therefore, interventions to reduce cardiovascular risk, including anti-hypertensive therapy, will be more effective if they could concomitantly ameliorate vascular endothelial dysfunction [7,8,9].

Epoxyeicosatrienoic acids (EETs), the metabolites of arachidonic acid synthesized by cytochrome P450 epoxygenases [10], are initially identified as endothelium-derived hyperpolarizing factors, with potent anti-hypertensive, anti-inflammatory, anti-apoptotic, and anti-proliferative properties [11]. However, they are rapidly degraded into the less active dihydroxyeicosatrienoic acids via soluble epoxide hydrolase (sEH) [12]. Therefore, the inhibition of sEH appears to be a potential approach for enhancing the bioavailability of EETs in various pathological conditions, including cardiovascular diseases [13,14,15]. An sEH inhibitor, 12-(3-adamantan-1-yl-ureido) dodecanoic acid, prevented endothelial dysfunction by restoring EETs-mediated relaxation in the coronary arteries of mice with renovascular hypertension [16]. The mice with the endothelium-specific expression of sEH (Tie2-sEH Tr) exhibited impaired endothelium-dependent vasodilation to acetylcholine in the cerebral circulation [17], whereas the inhibition of sEH with t-AUCB reversed the decreased coronary reactive hyperemia seen in Tie2-sEH Tr mice [18]. Studies show that the increased expression and activity of endothelial nitric oxide synthase (eNOS) that is stimulated by EETs may be related to the P13K/Akt and MAPK signaling pathways in cultured bovine aortic endothelial cells [19,20].

Although growing evidence indicates the impact of EETs on endothelial cell function, the molecular mechanism underlying the effects of EETs on endothelial nitric oxide synthase (eNOS) remains elusive. Small non-coding RNAs (miRNAs), which are generally non-coding RNAs, have been shown to play critical roles in hypertension and hypertension-associated endothelial dysfunction [21,22]. The NF-κB-responsive miR-31-5p decreased eNOS mRNA stability and, thus, led to the inhibition of eNOS expression and the subsequent suppression of NO/cGMP in TNFα-stimulated human umbilical vein endothelial cells [23]. MiR-217 resulted in diminished eNOS expression by down-regulating the vascular endothelial growth factor and apelin receptor pathways in the endothelial cells, from endothelial-specific inducible miR-217 C57BL/6J knock-in mice [24]. Exosomes derived from atorvastatin-pretreated mesenchymal stem cells stimulated the Akt/eNOS cascade, to augment the angiogenesis of endothelial cells by up-regulating miR-211-3p, thereby accelerating wound repair in diabetic rats [25]. In addition, one study revealed that increased EETs levels caused by a novel sEH inhibitor, TPPU, could improve the angiogenic function of endothelial progenitor cells by up-regulating miR-126 expression in myocardial infarction mice under exercise conditions [26]. Therefore, the current study aimed to investigate whether EETs elevation by the sEH inhibitor t-AUCB could improve vascular endothelial function by influencing miRNA expression in hypertensive rats and elucidate the underlying molecular mechanism.

## 2. Materials and Methods

### 2.1. Animals and the 2K1C Model

Male Sprague Dawley rats (SD, 6–8 weeks old) were supplied by the Experimental Animal Center of Peking University Health Science Center (PKUHSC). The rats were housed in the cages (Fengshi, Jiangsu, China) with sterile padding (Keao Xieli, Beijing, China). They had free access to the standard chow (Keao Xieli, Beijing, China) and water, unless specified elsewhere, at a constant temperature (25 ± 3 °C) under a 12 h light/12 h dark cycle. After the rats were anesthetized with ketamine/xylazine (75 and 6 mg/kg body weight, respectively), we performed a midline laparotomy to expose the left kidney. Two-kidney-one-clip (2K1C)-induced renovascular endothelial dysfunction, associated with hypertension, was applied using U-shaped silver clips. A clip was placed around the left renal artery, leaving a ~0.2 mm gap for blood flow. In the normotensive group (Sham), the rats underwent the same operation except for the occlusion of the left renal artery. This investigation was approved by the Animal Experimentation Ethics Committee of PKUHSC, and all the procedures were performed in accordance with the Guide for the Care and Use of Laboratory Animals, published by the US National Institutes of Health (NIH Publication, 8th Edition, 2011).

### 2.2. Systolic Blood Pressure Measurement

The systolic blood pressure (sBP) of the rats was determined using the tail-cuff method with the CODA high-throughput non-invasive blood pressure system (Product # CODA-HT8, Kent Scientific Corporation). The arterial pressures were measured at 0, 2, 4, 6, and 8 weeks after the 2K1C surgery. Eight weeks later, some of the hypertensive rats received t-AUCB (1 mg/kg/day) in their drinking water for 1 week. Thereafter, the sBP of the 3 group rats (Sham, 2K1C, and 2K1C + t-AUCB) were evaluated at the end of week 9.

### 2.3. Determination of Renal Blood Flow

Under anesthesia, a 2-cm abdominal incision was first made in the left flank, then the left kidney was exposed at a distance of 15 cm from a laser Doppler blood perfusion imager (PERIMED, PIM3, Stockholm, Sweden). After stabilization, the renal blood flow was measured and recorded at a rate of 5 images per second by the corresponding software (PIMSoft) (Stockholm, Sweden). The regions of interest (ROIs) were drawn over the left kidney to locate the blood flow. The ROIs were counted within 60 s to generate the signal intensity curves. Thereafter, the abdominal wall layer was sutured after the left kidney was returned. Then, the blood flow of the right kidney was examined. The perfusion unit (PU) is a basic index of laser Doppler measurement, which refers to the Doppler displacement value generated by red blood cells in flowing capillaries and is a relative unit by which to measure the microcirculation blood flow of local tissues in depth. The change in PU value directly reflects the change in the microcirculation blood flow of tissues.

### 2.4. Arterial Rings Preparation and Functional Assay

The rats were sacrificed by CO_2_ suffocation and the right intrarenal arteries were removed and placed in ice-cold Krebs solution. The arteries were cleaned of adhering tissue and were then cut into ring segments of ~1.5 mm in length. The arterial rings were suspended in a wire myograph (Danish Myo Technology, Aarhus, Denmark) for detecting endothelium-dependent relaxation with acetylcholine (ACh, 10**^−^**^8^ to 10**^−^**^5^ mol/L) and endothelium-independent relaxation with sodium nitroprusside (SNP, 10**^−^**^9^ to 10**^−^**^5^ mol/L), as previously reported [27].

### 2.5. Primary Culture of Rat Renal Artery Endothelial Cells

The intrarenal arteries of rat bilateral kidneys were dissected in sterilized PBS, under a stereoscopic microscope. After digestion by 0.2% type IA collagenase, dissolved in 5 mL sterilized PBS for 10 min at 37 °C, RPMI-1640 medium supplemented with 10% FBS, 100 IU penicillin, and 100 μg/mL streptomycin were added to terminate the digestion process. The endothelial cells were then collected by centrifugation (1000× *g* rpm × 5 min). Thereafter, the pellet was gently re-suspended in complete RPMI-1640 medium and cultured in a 35-mm culture dish. After 45 min of incubation, the medium was changed to remove the other cell types. Then, 24 h later, the medium was changed again. The primary cells were maintained until ~70% confluence was reached after 5~6 days. Next, the cells were transferred into 6-well plates, 12-well plates, or confocal dishes. Finally, 4~5 days later, we used the first passage cells to perform various experiments.

### 2.6. Adenoviral Infection

Arterial rings of the right kidneys or endothelial cells from the bilateral kidneys of 2K1C rats were infected with pAd-CA-AMPK, pAd-UCP2, or pAd-LAC-Z, and intrarenal arterial rings or endothelial cells from 2K1C + t-AUCB rats were infected with pAd-DN-AMPK, pAd-shUCP2, or pAd-scramble, using a protocol of 12 h exposure to 6 μL of adenovirus in a 6-well plate (1 × 10^8^ pfu/mL); then, we changed the medium. Finally, 48 h after infection, the arterial rings were ready for the functional assay and endothelial cells were collected for Western blot or qRT-PCR assays.

### 2.7. Measurements of EETs

Plasma was reserved from the Sham, 2K1C, and 2K1C + t-AUCB rats. The renal arteries and renal arterial endothelial cells from the 3 groups were lysed according to the kit instructions. Then the levels of EETs in plasma, the renal arteries, and endothelial cells were assayed using an EETs ELISA kit (Mlbio, Shanghai, China), according to the manufacturer’s instructions.

### 2.8. Treatment of Endothelial Cells 

Renal arterial endothelial cells from 2K1C or 2K1C + t-AUCB rats were seeded in 6-well or 12-well plates at ~70% confluence and then transfected with miR-155–5p inhibitor (100 nmol/L), mimics (50 nmol/L), or the corresponding negative control (NC, 100 or 50 nmol/L, respectively) using the Lipofectamine RNAiMax reagent (Invitrogen, Carlsbad, CA, USA). The MiR-155-5p inhibitor, its mimics, or the corresponding NC are four sequences that are synthesized chemically. The MiR-155-5p inhibitor could specifically target miR-155-5p and would result in a decrease in the amount of miR-155-5p in endothelial cells, while the miR-155-5p mimics could simulate endogenous miR-155-5p in cells and enhance the function of endogenous miR-155-5p. In addition, NC of miR-155-5p inhibitor or mimics did not affect the content of miR-155-5p in the cells, to eliminate false positives. Then, 12 h later, the medium was changed. The cells were then collected for Western blot analysis and qRT-PCR, 48 h after transfection. Renal arterial endothelial cells from 2K1C rats were cultured with the NF-κB inhibitor, pyrrolidinedithiocarbamic acid (PDTC, 100 μmol/L), a membrane-permeable radical scavenger, Tempol (1 μmol/L), the mitochondrial ROS scavenger coenzyme Q10 (20 μmol/L), or the NO donor SNP (20 μmol/L) for 24 h. Endothelial cells from 2K1C + t-AUCB rats were treated with NOS inhibitor L-NAME (100 μmol/L, 30 min). Endothelial cells from the Sham group were incubated with the NF-κB activator, betulinic acid (BA, 100 nmol/L), in the presence or absence of t-AUCB (1 μmol/L) for 24 h. Cells from Sham rats were also treated with ROS inducer, hypoxanthine (100 μmol/L), plus xanthine oxidase (0.01 U/mL), in the presence or absence of t-AUCB (1 μmol/L) for 24 h. After various treatments, the cells were harvested for Western blot analysis, qRT-PCR, or NO detection.

### 2.9. Western Blot Analysis

The isolated renal arteries or renal artery endothelial cells were homogenized in RIPA lysis buffer containing protease inhibitors and phosphatase inhibitors. Protein extraction and Western blot analysis were performed as previously described [27]. Antibodies against phospho-eNOS (Ser^1177^, 1:1000), eNOS (1:1000), phospho-AMPKα (Thr^172^, 1:1000), AMPKα (1:1000), NF-κB subunit p65 (1:1000), and UCP2 (1:1000) were obtained from Cell Signaling Technology (Beverly, MA, United States). The antibody against β-actin (1:5000) was obtained from Biodragon Technology (Beijing, China). Antibodies against IκBα (1:1000) and phospho-IκBα (Ser^36^, 1:1000) were purchased from ABclonal Technology (Wuhan, China). During the analysis of the data, we averaged all the data of Sham or Control rats. All the data, including every piece of Sham or Control data, were then compared with the average.

### 2.10. RNA Extraction and Quantitative Real-Time PCR

The total RNA was extracted from fresh renal arteries and endothelial cells, using TRIzol reagent (Invitrogen, CA, USA). The RNA concentration and quality were measured using a NanoDrop ND-2000 (Thermo Scientific, Waltham, MA, USA). The First Strand cDNA Synthesis Kit (Novoprotein, Shanghai, China) and the miDETECT A Track miRNA qRT-PCR Starter Kit (Ribobio, Guangzhou, China) were applied for reverse transcription. The mRNA levels of eNOS and miR-155-5p were detected via quantitative real-time qPCR, with the application of SYBR^®^ qPCR Super Mix (Novoprotein, Shanghai, China) and the miDETECT A Track miRNA qRT-PCR Starter Kit. All primers are listed in Table S1 in the Supplementary Materials.

### 2.11. ROS Detection Using Dihydroethidium and MitoSOX Staining

Dihydroethidium (DHE) (Beyotime Biotechnology, Shanghai, China) was used to detect the generation of ROS in the en face preparation of intrarenal arteries and endothelial cells. The arteries that were cut open and the cells that were seeded in confocal dishes were loaded with DHE at 150 μmol/L or 5 μmol/L for 20 min, respectively. MitoSOX (Invitrogen, Carlsbad, CA, USA) was applied to detect the mitochondrial ROS at 5 μmol/L for 15 min in the endothelial cells that were seeded in confocal dishes. ROS fluorescence was measured using the Leica Application Suite X (LAS X) (Leica, Wetzlar, Germany) at an excitation of 515 nm and an emission of 585 nm. The data were also analyzed using the LAS X software.

### 2.12. Total Nitric Oxide (NO) Production

Total NO production in the renal arteries and endothelial cells was determined by testing the concentrations of nitrate and nitrite, the stable metabolites of NO, using the Total Nitric Oxide Assay Kit (Beyotime Biotechnology), according to the manufacturer’s instructions.

### 2.13. Statistical Analysis

The results represent the mean ± SEM. The relaxation was expressed as a percentage of Phe-induced contraction. Data were analyzed using GraphPad Prism software (version 9.0, San Diego, CA, USA). The protein expression was quantified and analyzed by ChemiDoc XRS + (Bio-Rad Laboratories, Inc, Hercules, CA, USA). Statistical significance was determined by a one-way ANOVA, followed by Tukey’s test and the least significant difference (LSD) test for multiple comparisons. A two-way ANOVA was also applied when analyzing the relaxations. *p* < 0.05 was considered to be statistically significant.

## 3. Results

### 3.1. t-AUCB Restored Endothelial Function via Increasing EETs in 2K1C Hypertensive Rat Renal Arteries

**Figure 1:**
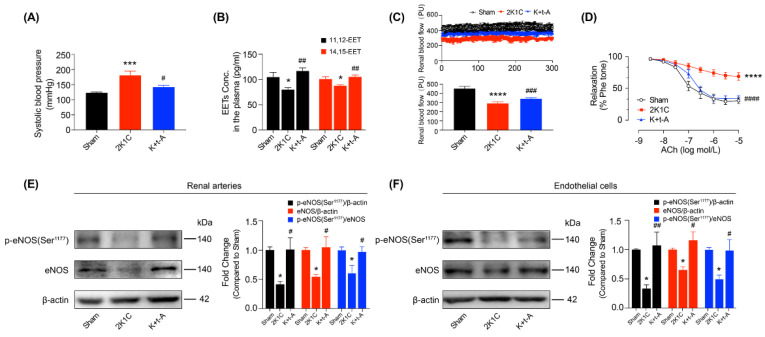
t-AUCB restored renal endothelial function via increasing the EETs in hypertensive rats. Systolic blood pressure (**A**) and EETs concentrations (**B**) in the plasma of Sham, 2K1C, and t-AUCB-treated 2K1C (K + t-A) rats. Blood flow of the right kidney in the three groups of rats (**C**). ACh-induced endothelium-dependent relaxation in the renal arteries from the three groups (**D**). The expression and phosphorylation of eNOS, as well as their ratio in the renal arteries (**E**) and endothelial cells (**F**). *n* = 4–11 per group. Data are presented as mean ± SEM. * *p* < 0.05, *** *p* < 0.001, **** *p* < 0.0001 vs. Sham; # *p* < 0.05, ## *p* < 0.01, ### *p* < 0.001, #### *p* < 0.0001, vs. 2K1C.

The two-kidney-one-clip (2K1C) operation progressively increased the systolic blood pressure (sBP) of rats and the sBP remained stable at week 8 (Figure S1A, Supplementary Materials). The experimental group of hypertensive rats treated with the soluble epoxide hydrolase (sEH) inhibitor, t-AUCB, demonstrated significantly reduced sBP, as shown in Figure 1A. sEH rapidly metabolizes the epoxyeicosatrienoic acids (EETs) into the less active dihydroxyeicosatrienoic acids. We ascertained that the levels of 11,12-EET and 14,15-EET were reduced in hypertensive rat plasma, but t-AUCB restored their concentrations (Figure 1B). The kidneys play an essential role in regulating arterial blood pressure. We demonstrated that the renal blood flow (RBF) was reduced in the right kidneys (Figure 1C) and the left kidneys (Figure S1B, Supplementary Materials) of hypertensive rats; t-AUCB restored the RBF of hypertensive rat right kidneys (Figure 1C) but failed in re-establishing the RBF of hypertensive rat left kidneys (Figure S1B). EETs have been shown to directly mediate smooth muscle relaxation [11]. Here, we found that the elevation of EETs by t-AUCB also improved the impaired endothelium-dependent relaxation (EDRs) to ACh (Figure 1D) but did not affect the endothelium-independent relaxation to SNP (Figure S1C) in 2K1C rat renal arteries. Moreover, the eNOS expression and phosphorylation, and the ratio of p-eNOS (Ser^1177^) and eNOS in both renal arteries (Figure 1E) and the renal arterial endothelial cells (Figure 1F) from hypertensive rats were diminished, which changes were reversed by t-AUCB. In addition, t-AUCB also increased the reduced levels of 11,12-EET and 14,15-EET in the renal arteries and endothelial cells from hypertensive rats (Figure S1D). These results indicate that the anti-hypertensive effect of EETs was partially mediated by the restoration of renal endothelial function and the subsequent recovery of renal blood flow.

### 3.2. t-AUCB Ameliorated Renal Arterial Endothelial Dysfunction by Stimulating AMPK in Hypertensive Rats

**Figure 2:**
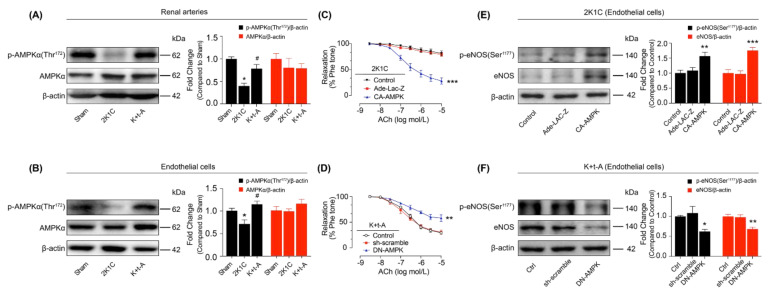
The role of AMPK in the improvement of renal endothelial function by t-AUCB in hypertensive rats. AMPK expressions and phosphorylation in the renal arteries (**A**) and in the endothelial cells (**B**) from Sham, 2K1C, and 2K1C + t-AUCB (K + t-A) groups. The relaxation response to ACh of the 2K1C rat renal arteries, over-expressed with constructively activated AMPK (CA-AMPK) (**C**), and those of 2K1C + t-AUCB rat arteries infected with dominant-negative AMPK (DN-AMPK) (**D**). The effects of CA-AMPK (**E**) or DN-AMPK (**F**) adenovirus on eNOS expressions and phosphorylation in the renal endothelial cells from 2K1C or 2K1C + t-AUCB rats. *n* = 5–8 per group. Data are presented as mean ± SEM. * *p* < 0.05, ** *p* < 0.01, *** *p* < 0.001 vs. Sham or the respective control; # *p* < 0.05 vs. 2K1C.

AMP-activated protein kinase (AMPK) plays an important role in vascular function, in part by activating eNOS. The phosphorylation of AMPK was reduced in the arteries (Figure 2A) and endothelial cells (Figure 2B) from 2K1C rat kidneys and was reversed by t-AUCB administration. The over-expression of AMPK restored the EDRs of the renal arteries (Figure 2C) and stimulated eNOS expression and phosphorylation (Figure 2E) in the renal endothelial cells from hypertensive rats. Moreover, the dominant-negative (DN) AMPK adenovirus inhibited the restoration of renal EDRs (Figure 2D) and the increase in eNOS expression and phosphorylation (Figure 2F) in the endothelial cells from hypertensive rats that were treated with t-AUCB. This finding suggested that t-AUCB ameliorated hypertensive renal arterial endothelial dysfunction by stimulating AMPK.

### 3.3. Uncoupling Protein 2 Mediated the Improvement of Endothelial Function, following AMPK Stimulation in Hypertensive Rats

**Figure 3:**
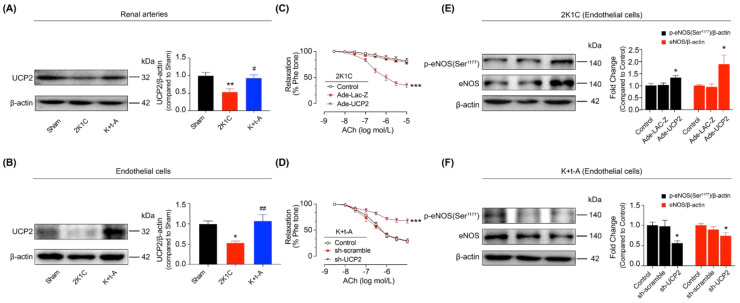
UCP2 mediated the improvement of endothelial function in hypertensive rats. Levels of UCP2 in the renal arteries (**A**) and the endothelial cells (**B**) from the three group rats. Impacts of over-expressing UCP2 (**C**) or sh-UCP2 (**D**) adenovirus on the endothelium-dependent relaxation of renal arteries from 2K1C or 2K1C + t-AUCB (K + t-A) rats. The expressions and phosphorylation of eNOS in the endothelial cells from 2K1C or 2K1C + t-AUCB rat renal arteries after the over-expression or knockdown of UCP2 (**E**,**F**). *n* = 4–7 per group. Data are presented as mean ± SEM. * *p* < 0.05, ** *p* < 0.01, *** *p* < 0.001 vs. Sham or the respective control; # *p* < 0.05, ## *p* < 0.01 vs. 2K1C.

Our previous study revealed that AMPK activation contributed to the amelioration of vascular endothelial dysfunction by upregulating the uncoupling protein 2 (UCP2) in spontaneously hypertensive rats (SHR) [28]. First, we also announced that the reduced UCP2 expression was raised by t-AUCB in the 2K1C rat renal arteries (Figure 3A) and renal endothelial cells (Figure 3B). The over-expression of UCP2 recovered the renal EDRs (Figure 3C) and increased eNOS expression and phosphorylation (Figure 3E) in renal endothelial cells from hypertensive rats. Moreover, the sh-UCP2 adenovirus suppressed these improvements by t-AUCB (Figure 3D,F). In 2K1C hypertensive rat renal endothelial cells, AMPK over-expression increased the UCP2 level, but UCP2 over-expression did not affect AMPK expression or activity (Figure S2A). In the endothelial cells from t-AUCB-treated hypertensive rats, the DN-AMPK adenovirus induced a reduction in UCP2 expression; however, the sh-UCP2 adenovirus did not affect AMPK expression or phosphorylation (Figure S2B). Based on the above observations, we concluded that t-AUCB resulted in the improvement of endothelial function through the activation of the AMPK/UCP2 cascade.

### 3.4. Uncoupling Protein 2 Protected the Endothelial Function by Suppressing ROS/NF-κB in Hypertensive Rat Renal Arteries 

**Figure 4:**
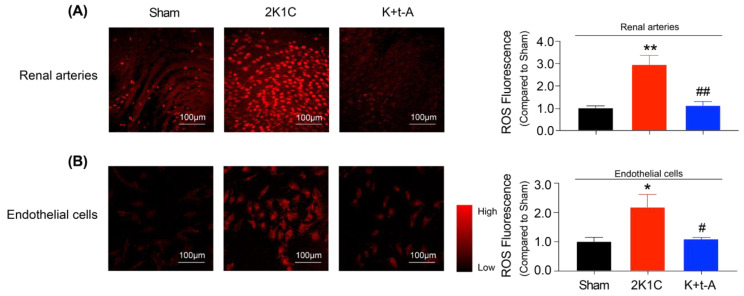
t-AUCB attenuated the oxidative stress in renal endothelial cells from hypertensive rats. Representative images ((**A**), **left**) of en face preparation for DHE fluorescence in the renal arterial endothelium of Sham, 2K1C, and 2K1C + t-AUCB (K + t-A) rats, along with the quantification of DHE fluorescence ((**A**), **right**). Representative images ((**B**), **left**) of ROS in the renal endothelial cells isolated from the three groups and the quantification of fluorescence ((**B**), **right**). *n* = 4 per group. Data are presented as mean ± SEM. * *p* < 0.05, ** *p* < 0.01 vs. Sham; # *p* < 0.05, ## *p* < 0.01 vs. 2K1C.

**Figure 5:**
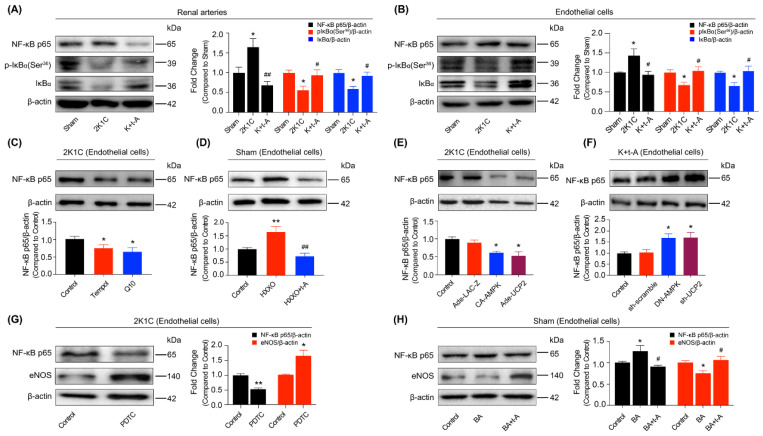
The suppression of NF-κB contributed to the amelioration of renal endothelial dysfunction induced by t-AUCB in hypertensive rats. The expression of NF-κB p65 and the expression and phosphorylation of IκBα (Ser^36^) in the renal arteries (**A**) and in the endothelial cells (**B**) from Sham, 2K1C, and 2K1C + t-AUCB (K + t-A) rats. The p65 expression in 2K1C hypertensive renal endothelial cells incubated with Tempol (1 μmol/L) and coenzyme Q10 (20 μmol/L) in 2K1C rat renal endothelial cells (**C**). The effect of t-AUCB on p65 expression in Sham rat renal endothelial cells treated with ROS inducer, 100 μmol/L hypoxanthine, plus 0.01 U/mL xanthine oxidase (HXXO) (**D**). The levels of p65 in the 2K1C rat endothelial cells infected with CA-AMPK or UCP2-expressing adenovirus (**E**) and in the 2K1C + t-AUCB rat endothelial cells under the treatment of DN-AMPK or sh-UCP2 adenovirus (**F**). Levels of p65 and eNOS in the 2K1C endothelial cells cultured with PDTC (100 μmol/L) (**G**). t-AUCB co-treatment to the Sham rat endothelial cells reduced the elevated p65 level and enhanced the down-regulated eNOS protein expression induced by NF-κB activator BA (100 nmol/L) (**H**). *n* = 4–9 per group. Data are presented as mean ± SEM. * *p* < 0.05, ** *p* < 0.01 vs. Sham or respective control; # *p* < 0.05, ## *p* < 0.01 vs. 2K1C, HXXO, or BA.

UCP2 negatively regulates mitochondrial ROS generation, thereby leading to the reduction of ROS in the cytoplasm. En face DHE staining demonstrated that the excessive accumulation of ROS was inhibited by t-AUCB in the hypertensive rat renal arterial endothelium (Figure 4A). Moreover, the cultured renal arterial endothelial cells from the three group rats exhibited similar changes (Figure 4B). In the meantime, we applied MitoSOX staining to detect mitochondrial ROS production in endothelial cells from the three group rats. The results showed that the mitochondrial ROS level was increased in the cells from hypertensive rats but that t-AUCB administration suppressed its generation (Figure S3). Next, we observed that the high levels of the NF-κB subunit p65 in the renal arteries (Figure 5A) and endothelial cells (Figure 5B) from hypertensive rats were also restrained by t-AUCB. After 24 h of incubation with a membrane-permeable radical scavenger, Tempol, or the mitochondrial ROS scavenger coenzyme Q10 down-regulated p65 expression in 2K1C hypertensive rat endothelial cells (Figure 5C) and improved the EDRs in hypertensive rat renal arteries (Figure S4A). Treatment with the ROS inducer hypoxanthine, plus xanthine oxidase for 24 h, resulted in up-regulated p65 expression in the renal endothelial cells (Figure 5D) and impaired EDRs in the renal arteries (Figure S4B) of Sham rats, which were both inhibited by co-incubation with t-AUCB (1 µmol/L). In addition, we verified that the over-expression of AMPK or UCP2 suppressed the p65 level in 2K1C hypertensive rat renal endothelial cells (Figure 5E), while DN-AMPK or the sh-UCP2 adenovirus elevated p65 expression in t-AUCB-treated hypertensive rat renal endothelial cells (Figure 5F). In order to further explore whether the increased expression of p65 was involved in eNOS down-regulation, we first treated the hypertensive rat endothelial cells with an NF-κB inhibitor, PDTC, and found that PDTC resulted in a decrease in p65 expression and an increase in eNOS expression in the renal arterial endothelial cells from hypertensive rats (Figure 5G) and the recovery of EDRs in the renal arteries from hypertensive rats (Figure S4A). Secondly, we announced that an NF-κB activator, BA, induced p65 up-regulation and eNOS down-regulation in the endothelial cells from Sham rat renal arteries (Figure 5H) and the suppression of EDRs in Sham rat renal arteries (Figure S4C), but t-AUCB co-treatment inhibited such effects (Figure 5H, Figure S4C). These results provided evidence that ROS/NF-κB mediated vascular endothelial dysfunction, while UCP2 contributed to the restoration of eNOS expression and EDRs by suppressing the ROS/NF-κB cascade in hypertensive rat renal arteries.

### 3.5. MiR-155-5p Was Involved in the Improvement of Renal Arterial Endothelial Function by EETs in Hypertensive Rats 

**Figure 6:**
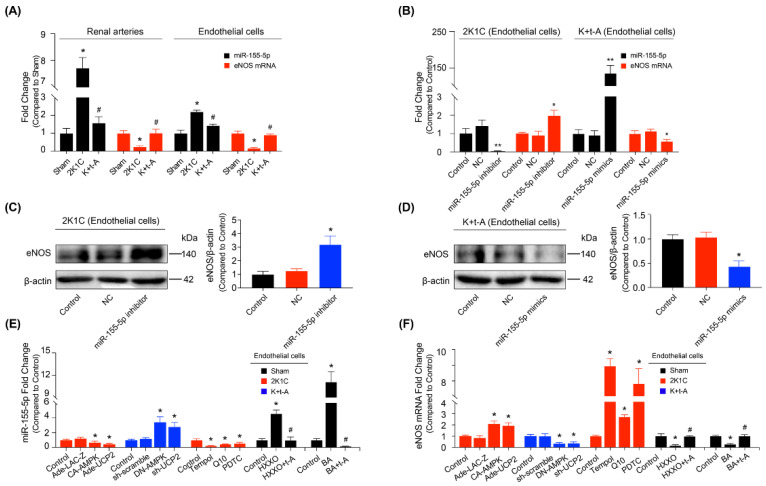
MiR-155-5p was involved in the regulation of eNOS. The miR-155-5p and eNOS mRNA levels in the renal arteries and endothelial cells from Sham, 2K1C, and 2K1C + t-AUCB (K + t-A) rats (**A**). The effects of the miR-155-5p inhibitor and mimics on the levels of miR-155-5p and eNOS mRNA in the renal endothelial cells from 2K1C or 2K1C + t-AUCB groups (**B**). The eNOS expression in the 2K1C rat renal endothelial cells transfected with miR-155-5p inhibitor (**C**), and that in 2K1C + t-AUCB rat endothelial cells treated with miR-155-5p mimics (**D**). The contents of miR-155-5p (**E**) and eNOS mRNA (**F**) in the three groups of rat renal endothelial cells under different treatments. *n* = 4–6 per group. Data are presented as mean ± SEM. * *p* < 0.05, ** *p <* 0.01 vs. Sham or respective control; # *p* < 0.05 vs. 2K1C, HXXO (hypoxanthine plus xanthine oxidase), or BA.

MiRNAs play key roles in the control of endothelial dysfunction in hypertension. The biogenesis of miR-155 or miR-155-5p response to NF-κB activation has been reported to be involved in eNOS down-regulation [29,30]. Herein, we revealed elevated miR-155-5p levels in the hypertensive rat renal arteries and renal arterial endothelial cells, accompanied by reduced eNOS mRNA expression, which were both reversed by t-AUCB (Figure 6A). The inhibitor of miR-155-5p could significantly suppress miR-155-5p levels and, thus, increase the levels of eNOS mRNA (Figure 6B) and protein expression (Figure 6C) in the hypertensive rat endothelial cells; however, the mimics of miR-155-5p clearly enhanced miR-155-5p expression and thereby reduced the eNOS mRNA level (Figure 6B) and protein expression (Figure 6D) in the t-AUCB-treated hypertensive rat endothelial cells. This part of the results suggested that miR-155-5p might mediate vascular endothelial dysfunction in hypertension, while t-AUCB improved endothelial function by suppressing miR-155-5p.

### 3.6. The AMPK/UCP2 Activation Regulated an NF-κB/miR-155-5p/eNOS/NO/IκB Cycle by Scavenging ROS in the Improvement of Endothelial Function 

**Figure 7:**
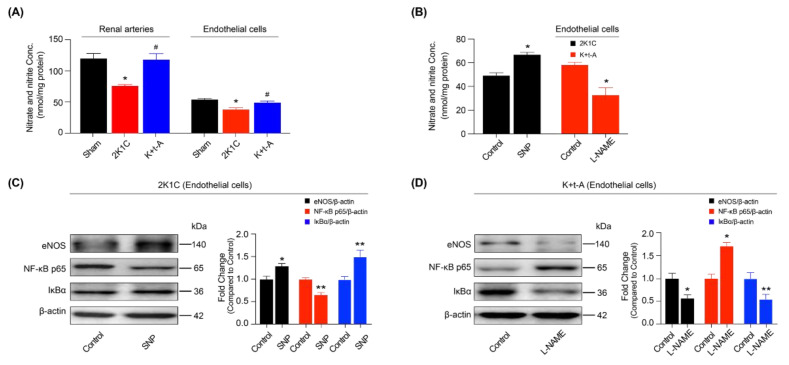
Modulation of nitric oxide (NO) on IκBα, NF-κB p65, and eNOS expression. The concentration of nitrate and nitrite in the renal arteries and endothelial cells (**A**). The effects of SNP or L-NAME on nitrate and nitrite concentrations in the endothelial cells, from 2K1C or 2K1C + t-AUCB (K + t-A) renal arteries (**B**). Expressions of IκBα, NF-κB p65, and eNOS in the 2K1C rat endothelial cells cultured with 20 μmol/L SNP for 24 h (**C**) and those in the 2K1C + t-AUCB rat endothelial cells treated with 100 μmol/L L-NAME for 30 min (**D**). *n* = 4–9 per group. Data are presented as mean ± SEM. * *p* < 0.05, ** *p* < 0.01 vs. Sham or the respective control; # *p* < 0.05 vs. 2K1C.

The following experiments investigated the regulatory mechanism of t-AUCB on miR-155-5p. AMPK or UCP2 over-expression down-regulated the miR-155-5p level (Figure 6E) and up-regulated eNOS mRNA expression (Figure 6F) in 2K1C hypertensive rat renal endothelial cells. On the other hand, DN-AMPK or sh-UCP2 adenovirus had the opposite effect in the t-AUCB-treated hypertensive rat endothelial cells (Figure 6E,F). The membrane-permeable radical scavenger, Tempol, mitochondrial ROS scavenger coenzyme Q10, and the NF-κB inhibitor PDTC all reduced miR-155-5p expression (Figure 6E) and increased eNOS mRNA level (Figure 6F) in the endothelial cells from hypertensive rats. To examine the regulatory actions of ROS and NF-κB on miR-155-5p, we treated the Sham rat endothelial cells with the ROS inducer hypoxanthine, plus xanthine oxidase (HXXO) or NF-κB activator BA, in the presence or absence of t-AUCB. The qRT-PCR results demonstrated that both HXXO and BA elevated the miR-155-5p level and reduced eNOS mRNA; such effects were reversed by co-incubation with t-AUCB (Figure 6E,F). Although the increase of miR-155-5p induced eNOS down-regulation, we then detected NO production. Our results showed that the reduced NO generation in hypertensive rats could be restored by t-AUCB treatment (Figure 7A). In addition, SNP incubation for 24 h increased NO level (Figure 7B), enhanced IκBα level, suppressed p65 expression, and elevated the eNOS protein level (Figure 7C) in hypertensive rat endothelial cells. However, applying the L-NAME treatment for 30 min diminished NO production (Figure 7B), reduced the expressions of eNOS and IκBα, and enhanced the level of p65 (Figure 7D) in t-AUCB-treated hypertensive rat endothelial cells. Furthermore, we demonstrated that t-AUCB treatment for 1 week rescued the down-regulated IκBα expression and phosphorylation in the renal arteries (Figure 5A) and endothelial cells (Figure 5B) from hypertensive rats. Taken together, an sEH inhibitor t-AUCB stimulated the AMPK/UCP2 cascade by elevating EETs; the activation of AMPK/UCP2 improved endothelial function by suppressing mitochondrial ROS production and, subsequently, regulating an NF-κB/miR-155-5p/eNOS/NO/IκB cycle in hypertensive rat renal arteries.

## 4. Discussion

Hypertension, a complex condition, is the leading cardiovascular risk factor. Vascular endothelial dysfunction is both a cause and a consequence of hypertension [31]. Patients with essential hypertension display impaired endothelium-dependent relaxations (EDRs) in the renal arteries [32]. Therefore, it is clinically important to develop appropriate interventions for effectively improving vascular endothelial dysfunction in patients with hypertension. Our present results demonstrated that two-kidney-one-clip (2K1C) surgery induced increased blood pressure and restrained renal EDRs in Sprague Dawley rats, while a soluble epoxide hydrolase (sEH) inhibitor, t-AUCB, effectively prevented such deleterious influences by elevating the levels of epoxyeicosatrienoic acids (EETs). The increased EETs attenuated ROS generation in endothelial cells in an AMPK/UCP2-dependent manner. Importantly, the amelioration of endothelial dysfunction was mostly related to the regulation of an NF-κB/miR-155-5p/eNOS/NO/IκB cycle, following ROS inhibition.

The afferent arteriolar relaxation response to acetylcholine in the rabbits depended, firstly, on endothelium-derived nitric oxide to act as soluble guanylate cyclase and, secondly, on 14,15-EET to stimulate the Ca^2+^-activated K^+^ channels [33]. Here, we revealed that the elevation of 11,12-EET and 14,15-EET by t-AUCB improved renal EDRs by modulating eNOS expression and activity in 2K1C hypertensive rat renal arteries. The inhibition of sEH attenuated high-fat diet (HDF)-mediated renal injury by partially activating the AMP-activated protein kinase (AMPK) [34]. EETs, plus an sEH inhibitor, AUDA, rescued the HDF- or palmitic acid-induced inhibition of AMPK and, thus, enhanced autophagy in mouse cardiomyocytes [35]. The activation of AMPK down-regulated NOx-derived ROS production in human renal arteries [36]. The AMPK-mediated up-regulation of uncoupling protein 2 (UCP2) was involved in the amelioration of cerebrovascular dysfunction in aging SD rats [37] and the improvement of renal endothelial function in SHRs [27]. UCP2 is a negative regulator of mitochondrial ROS generation and defends against oxidative stress [38]. Regarding the role of AMPK/UCP2 in improving vascular endothelial function, the above studies focused on its inhibitory effects on ROS and the subsequent restoration of NO bioavailability, while our results showed that in 2K1C rats under the therapy with t-AUCB, the suppression of ROS after AMPK/UCP2 activation was involved in modulating eNOS mRNA and protein expressions and enhancing eNOS phosphorylation at Ser^1177^. ROS could modulate an NF-κB response and NF-κB then targeted related genes, such as JNK, in turn, attenuating ROS [39]. Caffeic acid o-nitro phenethyl ester ameliorated myocardial ischemia/reperfusion injury, partly through inhibiting ROS generation via the suppression of NF-κB signaling [40]. The NOx-2/ROS/NF-κB axis was involved in oxidized high-density lipoprotein-induced endothelial injury [41]. To sum up, the activating effects of ROS/NF-κB in inducing endothelial dysfunction, as reported in the literature, were in accordance with our current results. Additionally, t-AUCB administration repressed the activation of ROS/NF-κB in hypertensive rat renal arterial endothelium.

As a nuclear transcription factor, NF-κB regulates the expression of numerous genes, including small non-coding RNAs (miRNAs). NF-κB played a role in down-regulating miR-376, as observed in the renal tubular cells in LPS-induced acute kidney injury mice [42]. NF-κB-responsive miR-31-5p decreased eNOS mRNA stability and resulted in the inhibition of eNOS expression in TNFα-stimulated human umbilical vein endothelial cells [23]. NF-κB activation impaired endothelial progenitor cell function via the biogenesis of miR-31 and miR-155 and the subsequent down-regulation of eNOS [29]. The induction of heme oxygenase-1 by Korean Red ginseng prevented endothelial senescence by inhibiting the NF-κB-dependent biogenesis of miR-155-5p, leading to eNOS down-regulation [30]. The current study announced that an increase in EETs restored eNOS mRNA level and protein expression, along with its phosphorylation by suppressing endothelial NF-κB/miR-155-5p, thus leading to the recovery of NO production and EDRs in the renal arteries of 2K1C hypertensive rats. The addition of the NO donors, S-nitrosoglutathione or SNP, significantly increased cytoplasmic IκBα, a major inhibitor of NF-κB, and concomitantly reduced the NF-κB binding activity in human aortic endothelial cells undergoing low shear stress [43]. On the one hand, we found that SNP incubation of the endothelial cells from hypertensive renal arteries elevated IκBα expression and reduced NF-κB expression. On the other hand, we demonstrated that an eNOS inhibitor, L-NAME, induced a decrease in IκBα and an increase in NF-κB in the renal arterial endothelial cells from hypertensive rats via t-AUCB treatment. Importantly, SNP or L-NAME also caused an increased or decreased eNOS expression in the above two types of endothelial cells, respectively. 

## 5. Conclusions

**Figure 8:**
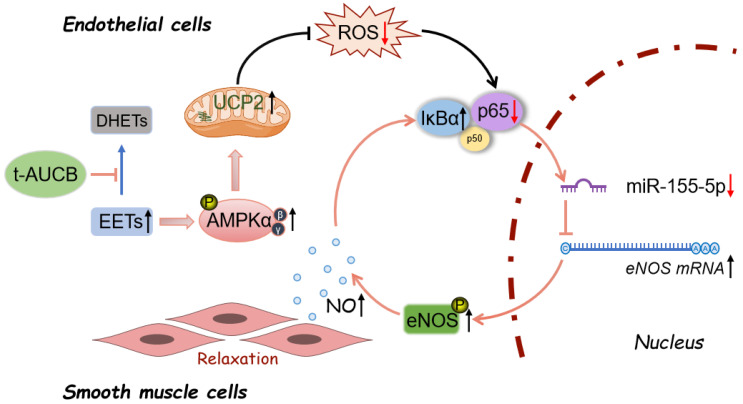
The proposed molecular mechanism for the protective effect of t-AUCB against endothelial dysfunction in hypertension. t-AUCB decreased the degradation of EETs and, thus, increased their levels. EETs stimulated AMPK/UCP2 activation, suppressed mitochondrial ROS production, and regulated an NF-κB/miR-155-5p/eNOS/NO/IκB cycle, thereby improving endothelial function in 2K1C hypertensive rat renal arteries.

In summary, we speculated that increasing EETs by t-AUCB attenuated renal arterial endothelial dysfunction via regulating an NF-κB/miR-155-5p/eNOS/NO/IκB cycle, after the activation of the AMPK/UCP2 cascade and the subsequent suppression of mitochondrial ROS production in hypertensive rats (Figure 8).

## Data Availability

The data presented in this study are available in this manuscript.

## Supplementary Materials

The following supporting information can be downloaded at: https://www.mdpi.com/article/10.3390/antiox11071372/s1. Figure S1: Evaluation of systolic blood pressure (sBP) and vascular function. Figure S2: AMPK mediated the up-regulation of UCP2. Figure S3: t-AUCB attenuated mitochondria-derived ROS in the renal endothelial cells from hypertensive rats. Figure S4: The endothelium-dependent relaxations (EDRs) in the 2K1C or Sham rat renal arteries under various treatments. Table S1: The primers of the miRNA and mRNA.
