# Supplementary material for: Soluble Epoxide Hydrolase Inhibitor t-AUCB Ameliorates Vascular Endothelial Dysfunction by Influencing the NF-κB/miR-155-5p/eNOS/NO/IκB Cycle in Hypertensive Rats
Source: Antioxidants (Basel). 2022 Jul 15;11(7):1372. doi: 10.3390/antiox11071372 (PMC9311992; doi:10.3390/antiox11071372)
Supplement: Supplementary file 1 [file antioxidants-11-01372-s001.zip › antioxidants-1769286-supplementary.pdf]

antioxidants

Supplementary Materials: Soluble Epoxide Hydrolase Inhibitor t-AUCB Ameliorates Vascular Endothelial Dysfunction by Influencing the NF-κB/miR-155-5p/eNOS/NO/IκB Cycle in Hypertensive Rats

Xiaorui Wang [1], Wenwen Han [1], Yi Zhang [1,2], Yi Zong [1], Na Tan [1], Yan Zhang [1,3], Li Li [1,3], Chang Liu [1] and Limei Liu [1,3,*]

[1] Department of Physiology and Pathophysiology, School of Basic Medical Sciences, Peking University, Beijing, 100191, China; 2011210004@bjmu.edu.cn (X.W.); 1911210009@pku.edu.cn (W.H.); 2111110083@bjmu.edu.cn (Y.Z.); 1911210056@bjmu.edu.cn (Y.Z.); tanna963@bjmu.edu.cn (N.T.); zhangy18@bjmu.edu.cn (Y.Z.); lilyby@bjmu.edu.cn (L.L.); 2111210001@stu.pku.edu.cn (C.L.)
[2] Department of Integration of Chinese and Western Medicine, School of Basic Medical Sciences, Peking University, Beijing, 102206, China
[3] Key Laboratory of Molecular Cardiovascular Science, Ministry of Education, Beijing, 100191, China
* Correspondence: liulm@bjmu.edu.cn; Tel.: +86-10-8280-1403

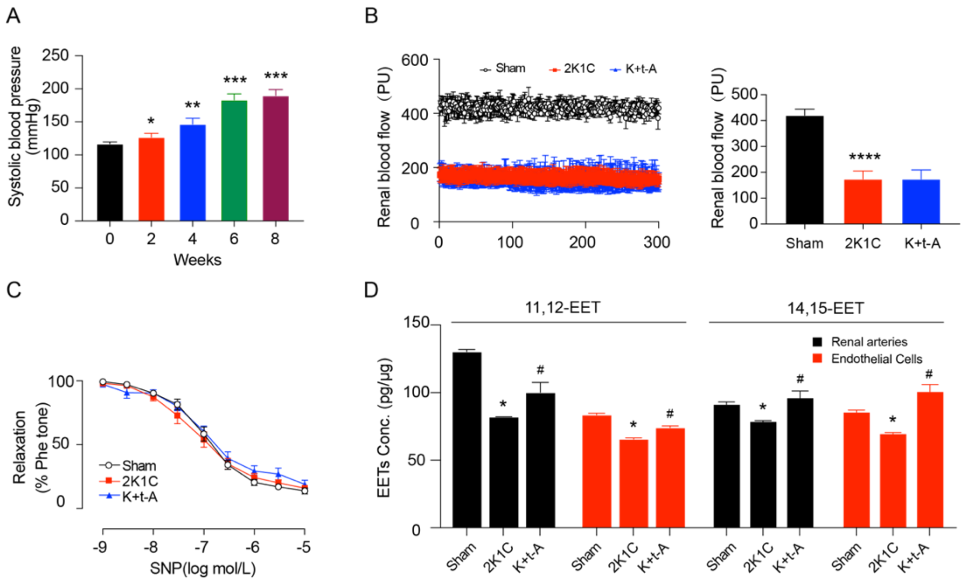

Figure S1. Evaluation of systolic blood pressure (sBP) and vascular function. The sBP of rats was recorded every two weeks (**A**). The renal blood flow of the rat left kidneys (**B**). The endothelium-independent relaxations response to SNP in the renal arteries from the three groups of rats (**C**). Levels of 11,12-EET and 14,15-EET in the renal arteries and endothelial cells (**D**). n = 9 for blood pressure measurement, n = 4–10 for other experiments. Data are presented as mean ± SEM. * $p < 0.05$, ** $p < 0.01$, *** $p < 0.001$, **** $p < 0.0001$ vs. 0 week or Sham; # $p < 0.05$ vs. 2K1C.

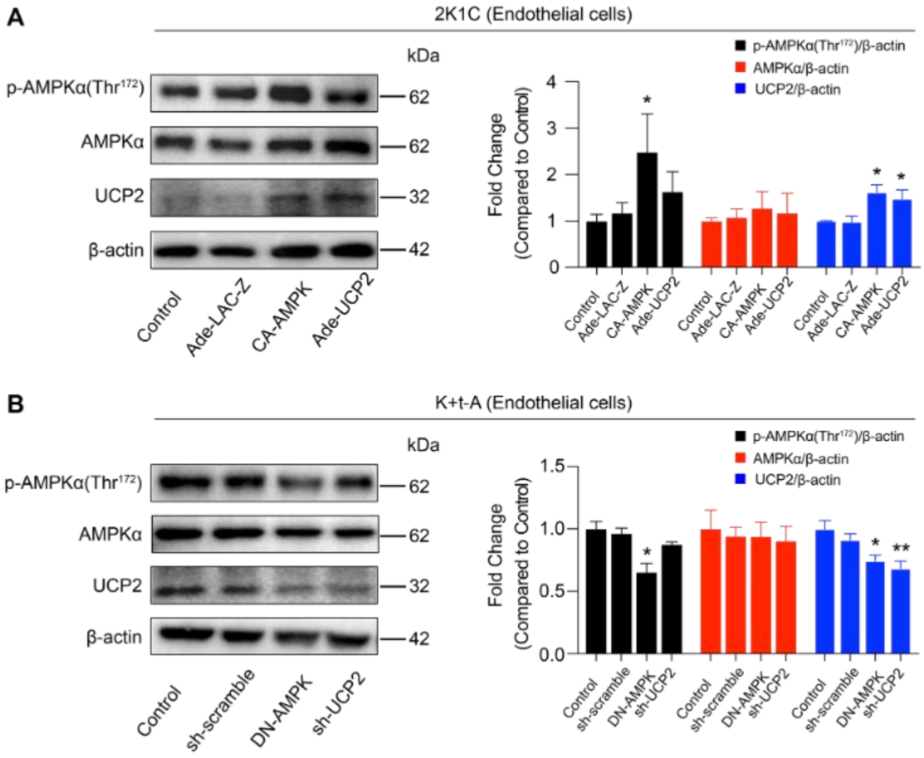

Figure S2. AMPK mediated the up-regulation of UCP2. The CA-AMPK adenovirus stimulated AMPK phosphorylation and elevated UCP2 levels without influencing AMPK expression, but the UCP2-expressing adenovirus increased UCP2 expression and did not affect AMPK expression and phosphorylation in the 2K1C rat renal endothelial cells (**A**). In the endothelial cells from hypertensive rats treated with t-AUCB (K + t-A), the DN-AMPK adenovirus reduced AMPK phosphorylation and UCP2 level, without affecting AMPK expression; however, sh-UCP2 adenovirus caused the down-regulation of UCP2 and had no effects on the expression and phosphorylation of AMPK (**B**). n = 5–7 per group. Data are presented as mean ± SEM. * $p < 0.05$, ** $p < 0.01$ vs. the respective control.

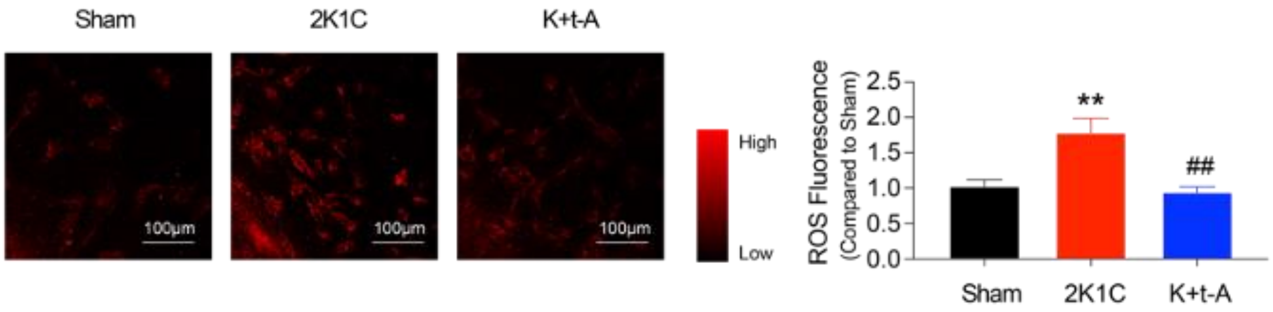

Figure S3. t-AUCB attenuated mitochondria-derived ROS in the renal endothelial cells from hypertensive rats. Representative images (left) of mitochondrial ROS production in the cells isolated from Sham, 2K1C, and 2K1C + t-AUCB (K + t-A) rats and quantification of fluorescence (right). n = 4–5 per group. Data are presented as mean ± SEM. ** $p < 0.01$ vs. Sham; ## $p < 0.01$ vs. 2K1C.

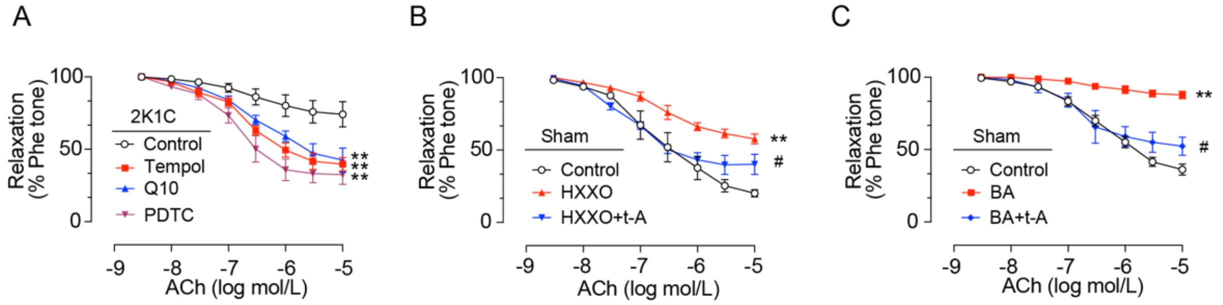

Figure S4. The endothelium-dependent relaxations (EDRs) in the 2K1C or Sham rat renal arteries under various treatments. The effects of the ROS scavenger, Tempol, the mitochondrial ROS scavenger coenzyme Q10, and the NF-κB inhibitor PDTC on the EDRs of 2K1C rat renal arteries (**A**). The ROS inducer hypoxanthine plus xanthine oxidase (HXXO) or the NF-κB activator BA impaired the EDRs in the renal arteries from Sham rats, which were both rescued by co-incubation with t-AUCB (**B**,**C**). $n = 4$–7 per group. Data are presented as mean ± SEM. ** $p < 0.01$ vs. respective control; # $p < 0.05$ vs. HXXO or BA.

Table S1. The primers of the miRNA and mRNA.

Gene Name	Gene ID		Primer Sequence (5′-3′)
miR-155-5p	102465831	Forward	CGTTAATGCTAATTGTGATAGGGGT
U6	120103225	Forward	TTGGAACGATACAGAGAAGATTAGCAT
eNOS	24600	Forward	CGAGATATCTTCAGTCCCAAGC
		Reverse	GTGGATTTGCTGCTCTCTAGG
Gapdh	24383	Forward	GTATGACTCCACTCACGGCAAA
		Reverse	GGTCTCGCTCCTGGAAGATG
